# SKP2 Regulates Pulmonary Inflammatory Injury Triggered by Bacterial Infection Through p27‐Mediated Degradation of FOXN3

**DOI:** 10.1002/mco2.70963

**Published:** 2026-09-11

**Authors:** Jinjin Yu, Xinghong Hu, Huanhuan Wang, Jingyu Yu, Lele Jin, Yingke Li, Jihong Zhou, Xinxing Zhu, Emmanuel Jairaj Moses, Chunfu Zheng, Yong Zhang, Wei Li

**Affiliations:** ^1^ Anhui Province Key Laboratory of Clinical and Preclinical Research in Respiratory Disease, Department of Respiratory and Critical Care Medicine First Affiliated Hospital Bengbu Medical University Bengbu China; ^2^ Regenerative Medicine Sciences Cluster, Advanced Medical and Dental Institute Universiti Sains Malaysia Kepala Batas Malaysia; ^3^ Molecular Diagnosis Center First Affiliated Hospital Bengbu Medical University Bengbu China; ^4^ Henan Joint International Research Laboratory of Stem Cell Medicine, School of Medical Engineering Xinxiang Medical University Xinxiang China; ^5^ Research Center of Clinical Laboratory Science, School of Laboratory Medicine Bengbu Medical University Bengbu China; ^6^ Department of Microbiology, Immunology & Infection Diseases University of Calgary Calgary Alberta Canada

**Keywords:** noncanonical NF‐κB signaling, p27^kip1^, phosphorylation, pulmonary inflammatory injury, SKP2

## Abstract

Excessive inflammatory responses are a primary driver of acute lung injury, yet the underlying molecular mechanisms remain incompletely understood. In this study, we identify SKP2, an E3 ubiquitin ligase, as a critical negative regulator of pulmonary inflammation during methicillin‐resistant *Staphylococcus aureus* (MRSA) infection. Mechanistically, SKP2 promotes the degradation of its direct substrate p27^kip1^ (p27), which functions as an activator of a noncanonical NF‐κB signaling pathway. We further demonstrate that p27 facilitates the recruitment of p38 kinase to heterogeneous nuclear ribonucleoprotein‐U (hnRNPU), leading to direct phosphorylation of the transcriptional repressor FOXN3 at serine residues 83 and 85. This dual phosphorylation event triggers subsequent proteasomal degradation of FOXN3, thereby relieving transcriptional repression and enabling NF‐κB–driven activation of pro‐inflammatory genes. In vivo, genetic ablation of *Skp2* worsens pulmonary inflammatory injury, elevates neutrophil infiltration, and significantly reduces survival in MRSA‐infected mice, and these effects are greatly rescued by concurrent p27 depletion. Collectively, our findings uncover the SKP2–p27–FOXN3 axis as a pivotal regulatory module in pulmonary inflammation and suggest that targeting this axis may offer a promising therapeutic strategy for bacterial infection‐induced lung injury.

## Graphical abstract

In response to MRSA infection, p27, a downstream effector protein of SKP2, helps recruit the kinase p38 to FOXN3. This interaction induces the degradation of FOXN3 through direct phosphorylation, leading to activation of the NF‐κB signaling pathway and ultimately triggering pulmonary inflammatory injury.

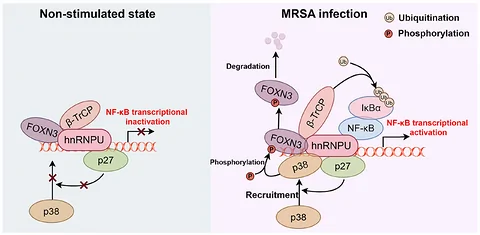

## 1 Introduction

As an essential pathway that mediates the inflammatory response, the NF‐κB signaling cascade plays a dominant role in the pulmonary inflammatory response to infection by a wide variety of pathogens, especially *Staphylococcus aureus* (*S. aureus*), by modulating the accumulation of downstream inflammatory factors. To alleviate excessive NF‐κB‐mediated inflammation and pulmonary injury, understanding the regulatory mechanisms leading to its transcriptional activation in pulmonary bacterial infection is necessary. The NF‐κB protein family includes RelA (p65), RelB, c‐Rel, p50/p105 (NF‐κB1), and p52/p100 (NF‐κB2), the latter of which is heterodimerized in its active state [1, 2, 3, 4, 5, 6]. The p65/p50 complex plays the most prominent role in inflammatory signal transduction and associated pathological processes.

Both canonical and noncanonical NF‐κB activation pathways have been extensively characterized over the past several decades [5, 7, 8, 9, 10, 11, 12, 13]. Under basal, unstimulated conditions, a substantial proportion of NF‐κB dimers remains inactive and is retained in the cytoplasm via high‐affinity binding to the major inhibitory protein IκBα [7, 14]. Upon proinflammatory signals, IKKβ‐mediated IκBα degradation releases NF‐κB for nuclear translocation and transcriptional activation [7, 15, 16, 17, 18]. In recent years, a noncanonical NF‐κB cascade independent of IKKβ has been identified in response to bacterial infection or lipopolysaccharide (LPS) stimulation [19]. In this pathway, IKKβ assists in recruiting heterogeneous ribonucleoprotein‐U (hnRNPU), which then forms the hnRNPU/β‐TrCP/IκBα complex. This complex subsequently triggers β‐TrCP‐mediated degradation of IκBα, leading to the activation of NF‐κB signaling. A recent study conducted by our group revealed that this noncanonical regulatory mechanism of NF‐κB activation is crucial for the pulmonary inflammatory response to various stimuli, notably methicillin‐resistant *Staphylococcus aureus* (MRSA) infection [20]. Therefore, exploring the molecular mechanisms behind this noncanonical NF‐κB signaling pathway activation could pave the way for significant advancements in the treatment of inflammatory disorders associated with bacterial infections.

SKP2 (S‐phase kinase‐associated protein 2) is a well‐characterized F‐box protein that serves as the substrate‐recognition subunit of the SCF (Skp1–Cullin–RBX1–F‐box) E3 ubiquitin ligase complex [21, 22]. Through its leucine‐rich repeats, SKP2 specifically recognizes and recruits target proteins for ubiquitination, marking them for proteasome‐dependent degradation. Its established substrates include the cyclin‐dependent kinase (CDK) inhibitor p27^Kip1^ (p27) and the epithelial cell adhesion molecule E‐cadherin, both of which are ubiquitinated by SKP2 and subsequently targeted to the proteasome for destruction [23, 24, 25, 26, 27, 28, 29, 30, 31]. p27, a member of the Cip/Kip family of CDK inhibitors, functions as a critical brake on cell cycle progression by binding to and inhibiting cyclin E–CDK2 and cyclin D–CDK4 complexes, thereby controlling G1‐to‐S phase transition. Beyond its canonical role in cell cycle regulation, accumulating evidence has implicated both SKP2 and p27 in the modulation of inflammatory signaling. For instance, altered SKP2 expression has been observed in inflammatory microenvironments, whereas p27 has been shown to influence macrophage activation and cytokine production [32, 33, 34]. However, the mechanistic intersection between the SKP2–p27 axis and NF‐κB signaling, particularly in the context of infectious inflammation, has remained largely elusive. While previous studies have separately linked SKP2 or p27 with NF‐κB‐mediated inflammatory responses [32, 33, 34], the precise molecular circuitry through which they cooperatively regulate NF‐κB activation—especially under bacterial challenge—is not yet defined.

In this study, our focus was on elucidating the essential role of the SKP2‒p27 axis in driving the activation of a noncanonical NF‐κB cascade. We found that p27 plays a significant role in the recruitment of the mitogen‐activated protein kinase (MAPK) p38 to hnRNPU. This recruitment leads to the degradation of FOXN3—a key inhibitor of the noncanonical NF‐κB cascade—through direct phosphorylation [20]. Functional studies indicated that the knockout (KO) of SKP2 exacerbated pulmonary inflammatory injury and reduced the survival rate of MRSA‐infected mice by increasing p27 abundance. This study highlights a potential role for the SKP2–p27 axis in regulating the pathological processes associated with MRSA infection‐induced acute pulmonary inflammatory injury through the modulation of FOXN3 stability. These findings suggest new targets for drug development aimed at treating pulmonary inflammatory conditions triggered by bacterial infections.

## 2 Results

### 2.1 KO of SKP2 Exacerbates Pulmonary Inflammatory Injury by Activating NF‐κB Signaling

**FIGURE 1:**
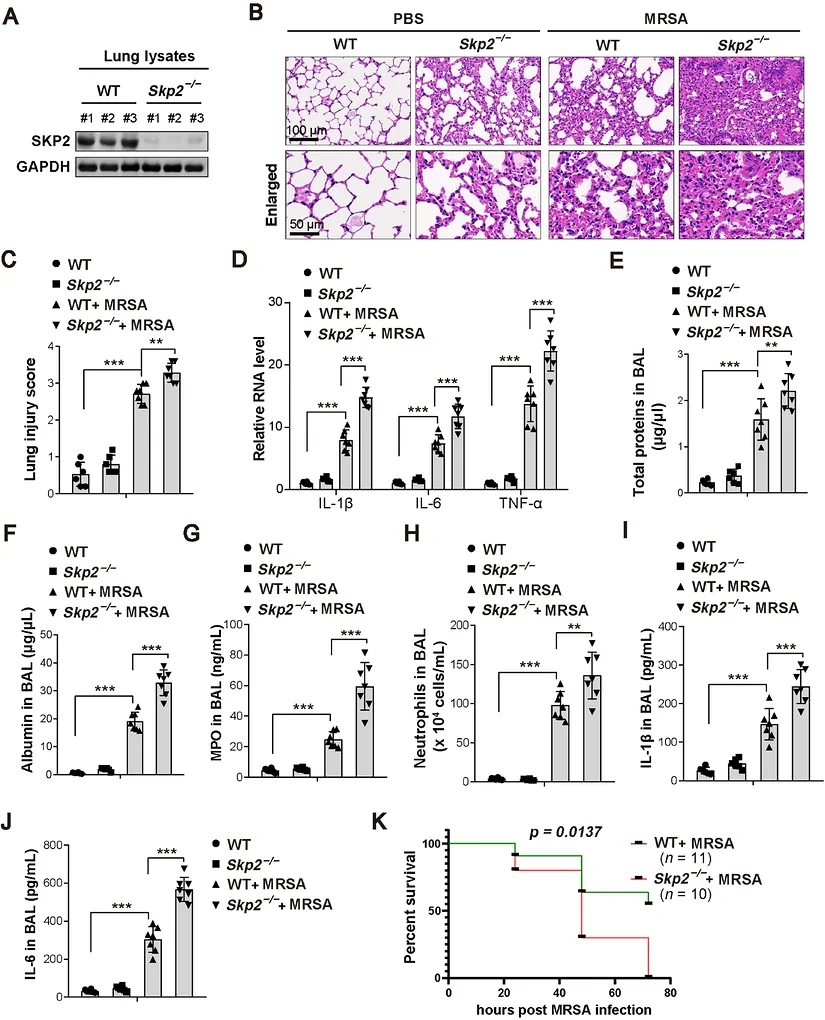
SKP2 knockout (KO) promotes the activation of pulmonary inflammatory injury. (A) Immunoblot analysis was performed to detect the protein level of SKP2 in the lung lysates of the mice with or without SKP2 KO. (B) H&E histological staining of lung sections from mice with or without SKP2 KO in the presence or absence of MRSA. (C) Scoring of pulmonary inflammatory injury shown in Figure 1B. (D) Quantitative PCR analysis was performed to examine the production of proinflammatory factors in the lungs of WT or SKP2 KO mice with or without MRSA infection (*n* = 6 for WT or *Skp2^−/−^*, *n* = 7 for WT+*S. aureus* or *Skp2^−/−^* +*S. aureus*). (E) Bicinchoninic acid (BCA) analysis of total protein concentrations in BAL fluid from WT or SKP2 KO mice with or without MRSA infection (*n* = 6 for WT or *Skp2^−/−^*, *n* = 7 for WT+*S. aureus* or *Skp2^−/−^* +*S. aureus*). (F) An enzyme‐linked immunosorbent assay (ELISA) was performed to measure the levels of albumin in BAL fluid from WT or SKP2 KO mice with or without MRSA infection (*n* = 6 for WT or *Skp2^−/−^*, *n* = 7 for WT+*S. aureus* or *Skp2^−/−^* +*S. aureus*). (G) Neutrophil enzyme MPO analysis of BAL fluid from WT or SKP2 KO mice with or without MRSA treatment (*n* = 6 for WT or *Skp2^−/−^*, *n* = 7 for WT+*S. aureus* or *Skp2^−/−^* +*S. aureus*). (H) Analysis of neutrophil counts in BAL fluid from WT or SKP2 KO mice with or without MRSA administration via Giemsa staining (*n* = 6 for WT or *Skp2^−/−^*, *n* = 7 for WT+*S. aureus* or *Skp2^−/−^* +*S. aureus*). (I and J) ELISA analysis was conducted to determine the levels of IL‐1β (I) or IL‐6 (J) in BAL fluid from WT or SKP2 KO mice with or without MRSA infection (*n* = 6 for WT or *Skp2^−/−^*, *n* = 7 for WT+*S. aureus* or *Skp2^−/−^* +*S. aureus*). (K) The survival rates of WT and SKP2 KO mice were monitored for 72 h after MRSA infection. The data C–J were assessed via one‐way ANOVA, and the data K were assessed via two‐tailed Student's *t*‐test. All the data are shown as the means ± SDs. ns, not significant; **p < 0.01 and ***p < 0.001 for comparisons between the indicated groups.

The precise role of the F‐box protein SKP2 in pulmonary inflammatory disorders remains unclear despite its established involvement in modulating the inflammatory response [35]. To investigate the potential regulatory role of SKP2 in pulmonary inflammatory injury, we generated SKP2 KO mice via CRISPR/Cas9‐mediated genome editing (Figure 1A). MRSA was administered intratracheally to 8‐week‐old *Skp2^−/−^* mice (SKP2‐KO) and their wild‐type (WT) littermates to induce pulmonary inflammatory injury. Twenty‐four hours after infection, hematoxylin and eosin (H&E) histological staining of lung samples revealed a significantly greater influx of neutrophils and more pronounced alveolar wall thickening in SKP2‐deficient mice than in WT controls (Figure 1B,C). Furthermore, quantitative PCR analysis confirmed that the expression levels of proinflammatory factors were significantly greater in the lung tissues of SKP2‐KO mice than in those of WT mice (Figure 1D). To further investigate the effects of SKP2 disruption on pulmonary inflammation, we isolated bronchoalveolar lavage (BAL) fluid from both WT and SKP2‐KO mice to compare the permeability of lung alveolar cells. As anticipated, the permeability of total proteins and albumin in the alveoli was significantly greater in SKP2‐KO mice than in WT mice (Figure 1E,F). This observation was consistent with elevated myeloperoxidase (MPO) activity, increased neutrophil counts, and increased secretion of proinflammatory factors following SKP2 KO (Figure 1G–J). Additionally, we evaluated the survival rates of WT and SKP2‐KO mice after MRSA infection. Importantly, our results indicated that SKP2 deletion resulted in a decreased survival rate in mice challenged with MRSA (Figure 1K). Collectively, these findings underscore the critical role of SKP2 in mitigating MRSA‐induced pulmonary inflammatory injury in mice.

**FIGURE 2:**
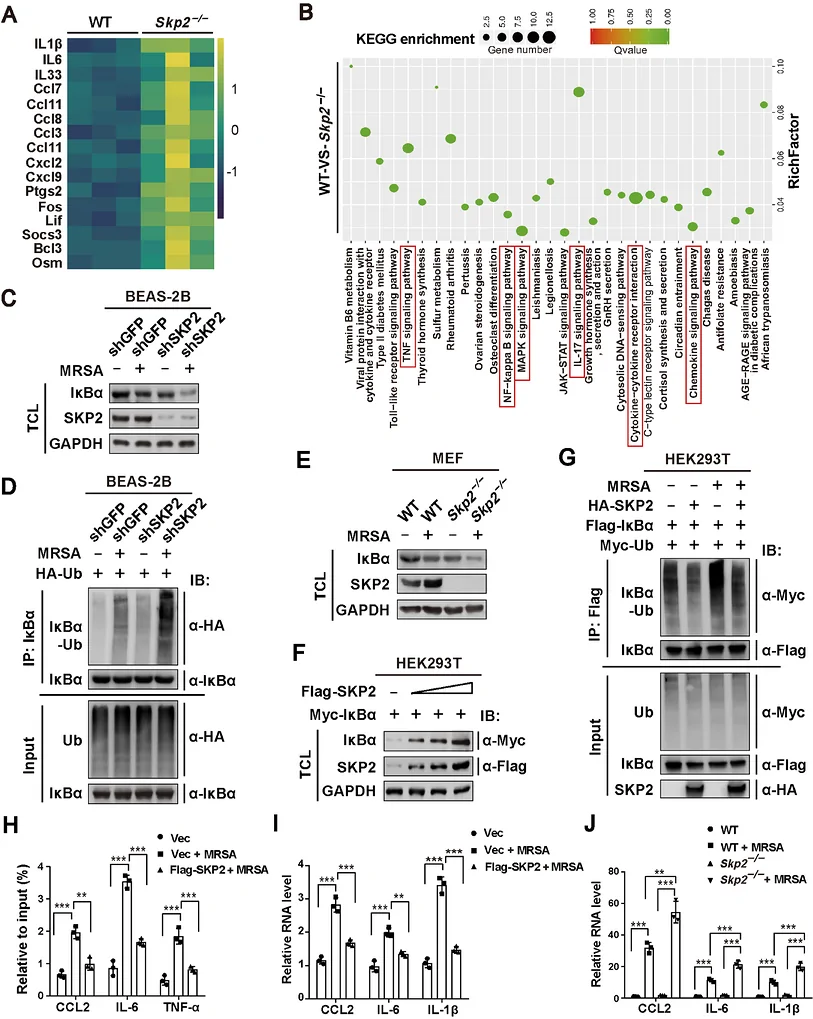
SKP2 negatively regulates NF‐κB signaling activation. (A) Representative heatmap showing the changes in the expression of NF‐κB‐regulated genes in lung lysates after SKP2 KO. (B) KEGG analysis showing the inflammatory pathways tightly modulated by SKP2. (C) Immunoblot analysis was performed to detect the effect of SKP2 knockdown on IκBα protein levels in BEAS‐2B cells in the presence or absence of MRSA infection (MOI, 20). TCL: total cell lysate; MOI: multiplicity of infection. (D) A ubiquitination assay was performed to assess the effect of SKP2 knockdown on IκBα ubiquitination in BEAS‐2B cells with and without MRSA infection. The cells were treated with MG‐132 (20 µM, 4 h) before collection. (E) Immunoblot analysis of IκBα in WT or *Skp2^−/−^* MEFs was performed to detect the effect of SKP2 KO on IκBα protein levels in the presence or absence of MRSA infection (MOI, 20). (F) Immunoblot analysis was conducted on HEK293T cells treated with 100 µg/mL cycloheximide (CHX) for 4 hours to assess the impact of incrementally elevated expression levels of Flag‐tagged SKP2 on Myc‐tagged IκBα protein expression. (G) A ubiquitination assay was performed to assess the effect of HA‐tagged SKP2 overexpression on Flag‐tagged IκBα ubiquitination in HEK293T cells with or without MRSA infection (MOI, 20). The cells were treated with MG‐132 (20 µM, 4 h) before collection. (H) A chromatin immunoprecipitation assay was performed to examine the effect of SKP2 on p65 binding affinity with its target gene promoters in BEAS‐2B cells with or without lentivirus‐mediated SKP2 overexpression in the presence or absence of MRSA treatment (MOI, 20). Vec: empty vector. (I) Quantitative PCR analysis was performed to examine the effects of SKP2 on proinflammatory factors in BEAS‐2B cells with or without lentivirus‐mediated SKP2 overexpression in the presence or absence of MRSA treatment (MOI, 20). (J) The production of proinflammatory factors in WT or *Skp2^−/−^* MEFs with or without MRSA treatment (MOI, 20) was measured via quantitative PCR analysis. The H–J data were assessed via one‐way ANOVA. All the data are shown as the means ± SDs. **p < 0.01 and ***p < 0.001 for comparisons between the indicated groups.

To explore the potential mechanisms underlying the suppressive role of SKP2 in MRSA‐induced pulmonary inflammation, we conducted RNA sequencing analysis of the transcriptomic profiles of the lungs of SKP2 KO and WT mice. Notably, the downstream regulatory targets of NF‐κB were expressed at significantly higher levels in SKP2 KO mice than in WT mice (Figure 2A). Furthermore, Kyoto Encyclopedia of Genes and Genomes (KEGG) analysis revealed that NF‐κB signaling and closely related inflammatory pathways were enriched in SKP2 KO mice (Figure 2B), which is consistent with our earlier qPCR data shown in Figure 1D. We further investigated the influence of SKP2 on the stability of the NF‐κB inhibitory protein IκBα in human bronchial epithelial (BEAS‐2B) cells and primary mouse embryonic fibroblasts (MEFs) under both uninfected and MRSA‐infected conditions. Immunoblotting and ubiquitination assays indicated that either shRNA‐mediated SKP2 knockdown or SKP2 KO notably accelerated ubiquitin‐mediated IκBα degradation (Figure 2C–E). In contrast, aberrant SKP2 overexpression resulted in increased IκBα stability by blocking its ubiquitination (Figure 2F,G). To further validate the role of SKP2 in suppressing NF‐κB transcriptional activation, we conducted a chromatin immunoprecipitation (ChIP) assay using an antibody against p65, a subunit of NF‐κB. Our results showed that SKP2 overexpression significantly inhibited MRSA‐induced recruitment of NF‐κB to its transcriptional targets (Figure 2H), ultimately impeding NF‐κB‐mediated transcriptional activation (Figure 2I). This finding is consistent with the elevated expression levels of NF‐κB target genes following SKP2 disruption (Figure 2J). In conclusion, our findings strongly suggest that SKP2 acts as a negative regulator of NF‐κB signaling activation.

### 2.2 SKP2 Regulates Noncanonical NF‐κB Signaling Activation by Controlling p27 Stability

**FIGURE 3:**
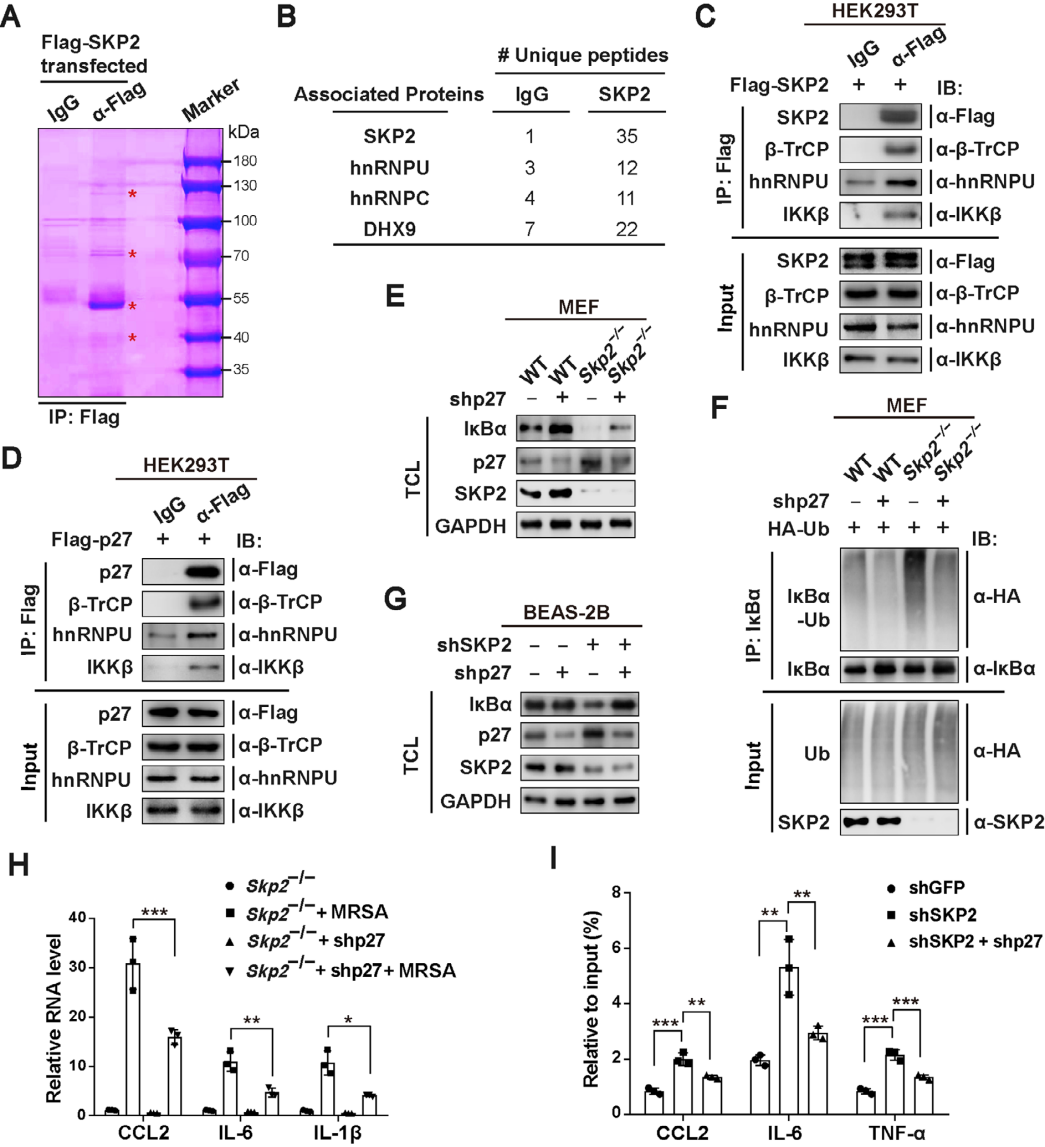
p27 depletion impedes SKP2 KO‐induced NF‐κB activation. (A) Coomassie brilliant blue staining analysis of immunoprecipitates affinity purified from HEK293T cells by anti‐Flag immunoprecipitation (IP). The red asterisk‐indicated bands were isolated for subsequent mass spectrometry analysis. (B) The red asterisk‐indicated bands from (A) were subjected to mass spectrometry analysis to define the unique SKP2‐associated peptides. (C) An anti‐Flag IP assay was performed to detect SKP2 interaction partners in HEK293T cells. (D) An anti‐Flag IP assay was performed to detect p27 interaction partners in HEK293T cells. (E) Immunoblot analysis of IκBα in WT or *Skp2^−/−^* MEFs with or without lentivirus‐mediated p27 knockdown in the presence of CHX (100 µg/mL, 4 h). The cells were selected with puromycin (2 µg/mL) for 3 days. (F) IκBα ubiquitination in WT or *Skp2^−/−^* MEFs infected with the indicated combinations of p27 shRNA and HA‐tagged ubiquitin was measured via a ubiquitination assay. The cells were selected with puromycin (2 µg/mL) for 3 days and then treated with MG‐132 (20 µM, 4 h) before collection. (G) An immunoblot assay was conducted to assess the levels of IκBα protein in BEAS‐2B cells with or without lentivirus‐mediated SKP2 or p27 knockdown, as well as their combined knockdown. The cells were treated with CHX (100 µg/mL, 4 h) before collection. (H) Quantitative PCR analysis showing the RNA expression levels of proinflammatory factors in WT or *Skp2^−/−^* MEFs with or without lentivirus‐mediated p27 depletion in the presence or absence of MRSA infection (MOI, 20). The cells were selected with puromycin (2 µg/mL) for 3 days before MRSA treatment. (I) A chromatin immunoprecipitation assay was conducted to demonstrate the binding affinity of p65 for its target gene promoters in BEAS‐2B cells infected with lentivirus expressing SKP2 shRNA, either alone or in combination with p27 shRNA or a negative control with a GFP shRNA, in the presence of MRSA infection (MOI, 20). The H and I data were assessed by one‐way ANOVA and are shown as the means ± SDs. *p < 0.05, **p < 0.01, and ***p < 0.001 for comparisons between the indicated groups.

To investigate the precise molecular basis by which SKP2 regulates NF‐κB pathway activation, we expressed and purified Flag‐tagged SKP2 in HEK293T cells and identified candidate interaction partners via mass spectrometry. This analysis identified the nuclear regulator hnRNPU, which is known to activate a noncanonical NF‐κB signaling cascade [20], along with its established interaction partners, as an apparent binding partner with SKP2 (Figure 3A,B). We then conducted coimmunoprecipitation (co‐IP) assays to verify that SKP2 associates with hnRNPU, as well as its interaction partners, IKKβ and β‐TrCP (Figure 3C). These findings strongly indicate that SKP2 might play a significant role in modulating the noncanonical NF‐κB signaling pathway.

In light of previous reports showing the involvement of p27, a well‐known substrate of the E3 ligase SKP2, in the regulation of the NF‐κB‐mediated inflammatory response [23, 33, 34], we hypothesized that the inhibition of the noncanonical NF‐κB cascade by SKP2 might also involve p27. To explore this possibility, we first examined the effects of p27 on NF‐κB pathway activation in HEK293T and BEAS‐2B cells in which p27 was knocked down via shRNA silencing. Immunoblotting revealed that p27 depletion strongly attenuated the ubiquitin‐dependent degradation of IκBα (Figure S1A,B) and subsequently impeded the MRSA‐induced recruitment of NF‐κB to its target gene promoters for transcriptional activation (Figure S1C,D). In line with these results, p27 overexpression resulted in increased ubiquitin‐mediated IκBα degradation (Figure S1E–G), suggesting that p27 functions as a potential activator of NF‐κB signaling. To further assess whether p27 was engaged in SKP2‐mediated noncanonical NF‐κB activation, we performed co‐IP assays and found that p27 was readily associated with huRNPU and its known interaction partners (Figure 3D). We subsequently silenced p27 using shRNA in MEFs isolated from our *Skp2^−/−^* mice to determine whether p27 plays a role in regulating the IκBα instability associated with SKP2 KO. Notably, p27 knockdown attenuated the increase in IκBα ubiquitination and proteasomal degradation associated with SKP2 KO (Figure 3E,F and Figure S2A), which suggested that p27 is a downstream effector of SKP2‐modulated NF‐κB pathway inactivation. Moreover, this observation was further confirmed in BEAS‐2B cells via combined p27 and SKP2 shRNA knockdown experiments (Figure 3G). Consistent with these findings, the transcriptional activation of NF‐κB triggered by SKP2 disruption was largely abolished in cells with p27 depletion (Figure 3H,I and Figure S2B,C). Indeed, the collective findings from our study strongly support the role of SKP2 as a critical inhibitor of noncanonical NF‐κB signaling through the regulation of the stability of its direct substrate, p27.

### 2.3 p27 Is Essential for the Phosphorylation‐Dependent Degradation of FOXN3 During the Activation of NF‐κB Signaling

**FIGURE 4:**
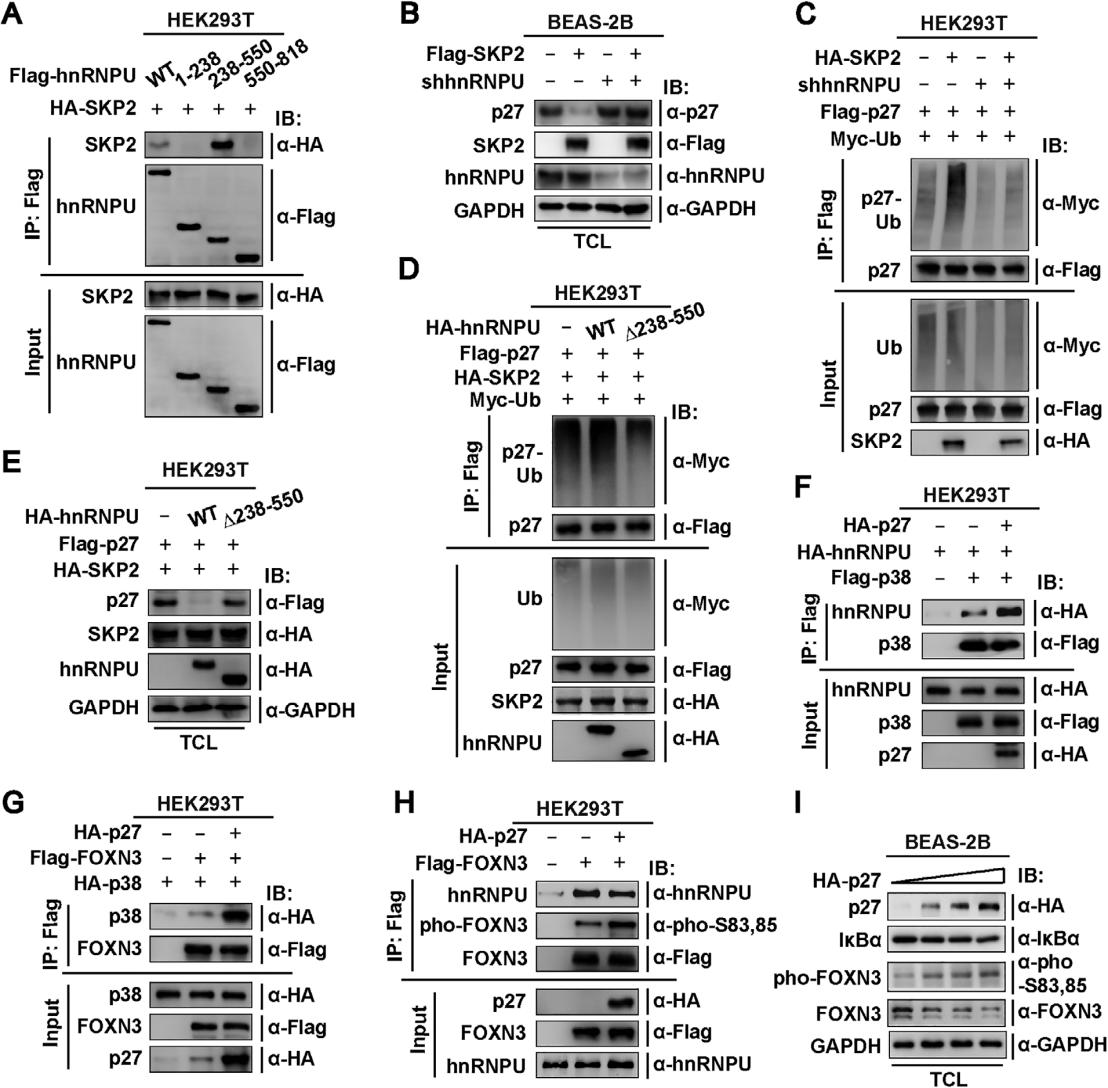
p27 helps recruit p38 to hnRNPU to facilitate phosphorylation‐dependent FOXN3 degradation and NF‐κB signaling activation. (A) Anti‐Flag IP was performed to define the region within hnRNPU responsible for its interaction with SKP2 in HEK293T cells transfected with various Flag‐tagged hnRNPU truncation peptides and HA‐tagged SKP2 as indicated. (B) Immunoblot analysis was performed to detect the effect of hnRNPU knockdown on SKP2‐mediated p27 degradation. BEAS‐2B cells were infected with lentivirus expressing a combination of Flag‐tagged SKP2 and hnRNPU shRNA or a control GFP shRNA. Prior to collection, the cells were treated with MRSA infection (MOI, 20). (C) A ubiquitination assay was performed to examine the effect of hnRNPU knockdown on SKP2‐mediated p27 ubiquitination in HEK293T cells transfected with the indicated combinations of HA‐tagged SKP2, Flag‐tagged p27, Myc‐tagged ubiquitin, and shhnRNPU. The cells were treated with MG‐132 (20 µM, 4 h) before collection. (D) A ubiquitination assay was performed to examine the effect of the lack of the hnRNPU middle region on p27 ubiquitination in HEK293T cells transfected with the indicated combinations of HAtagged SKP2, Flagtagged p27, Myctagged ubiquitin, and either HAtagged hnRNPU or its truncation mutant (Δ238–550). The cells were treated with MG‐132 (20 µM, 4 h) before collection. (E) Immunoblot analysis was performed to detect the effect of the lack of the hnRNPU middle region on p27 protein expression in HEK293T cells transfected with the indicated combinations. Prior to collection, the cells were treated with MRSA infection (MOI, 20). (F) Anti‐Flag IP was performed to detect the effect of p27 overexpression on the interaction between p38 and hnRNPU in HEK293T cells transfected with the indicated combinations of HA‐tagged p27, HA‐tagged hnRNPU, and Flag‐tagged p38. (G) Anti‐Flag IP was performed to detect the effect of p27 overexpression on the interaction between p38 and FOXN3 in HEK293T cells transfected with the indicated combinations of HA‐tagged p27, HA‐tagged p38, and Flag‐tagged FOXN3. The cells were treated with MG‐132 (20 µM, 2 h) before collection. (H) Anti‐Flag IP was performed to detect the effects of HA‐tagged p27 overexpression on FOXN3 S83 and S85 phosphorylation and its association with hnRNPU in HEK293T cells transfected with the indicated combinations. The cells were treated with MG‐132 (20 µM, 2 h) before collection. (I) Immunoblot analysis was conducted to detect the levels of the indicated proteins in BEAS‐2B cells expressing different doses of the p27 protein via lentiviral infection. The cells were treated with MRSA infection (MOI, 20) before collection.

Given the evident role of the SKP2–p27 axis in controlling noncanonical NF‐κB signaling, which drives the pulmonary inflammatory response to bacterial infection, our subsequent investigation focused on elucidating the mechanisms that regulate NF‐κB signaling during lung infection. In our previous publication, we identified hnRNPU as a scaffold protein involved in activating the noncanonical NF‐κB signaling pathway through domain‐dependent interactions [36]. On the basis of these findings, we hypothesize that hnRNPU may be involved in SKP2‐mediated p27 degradation. To investigate this further, we aimed to determine the specific region of hnRNPU that is essential for binding to either SKP2 or p27. Deletion mapping experiments indicated that a middle region within hnRNPU (amino acids 238–550) was required for its association with both SKP2 and p27 (Figure 4A and Figure S3A). Furthermore, through immunoblot and ubiquitination assays, we observed that knockdown of hnRNPU significantly inhibited SKP2‐mediated ubiquitination and subsequent degradation of p27 (Figure 4B,C). Interestingly, while hnRNPU depletion led to inhibition of p27 degradation, it also suppressed IκBα degradation and NF‐κB transcriptional activation (Figure S3B,C). This finding is consistent with the previously reported function of hnRNPU as a scaffold protein that supports β‐TrCP‐mediated IκBα degradation [19]. Consistently, aberrant hnRNPU overexpression resulted in the accelerated ubiquitination and degradation of p27 catalyzed by SKP2 (Figure S3D,E). Notably, the hnRNPU^Δ238‐550^ variant lacking the interaction site was strongly resistant to p27 ubiquitination and degradation by SKP2 (Figure 4D,E). These observations indicate that hnRNPU is necessary for the ubiquitination and subsequent degradation of p27 by SKP2.

To determine the biochemical function of p27 in hnRNPU‐mediated activation of NF‐κB signaling in pulmonary inflammatory injury, we first investigated the role of p27 in the interaction between hnRNPU and IKKβ or β‐TrCP, both of which are known to modulate the activation of NF‐κB signaling. Co‐IP experiments revealed that p27 overexpression did not appear to affect hnRNPU association with either IKKβ or β‐TrCP (Figure S3F,G). Building upon our previous report indicating that p38 can directly phosphorylate the NF‐κB inhibitory protein FOXN3 at the S83 and S85 residues, leading to its destabilization and consequent activation of noncanonical NF‐κB signaling [20], we proceeded to investigate the potential involvement of p27 in recruiting p38 to the hnRNPU/FOXN3 complex. Interestingly, we observed an increased interaction between hnRNPU and p38 under p27 overexpression (Figure 4F). The loss of the middle region (aa 238–550) of hnRNPU, which is responsible for its interaction with p27, significantly abrogated the hnRNPU‐p38 interaction (Figure S4A). Accordingly, the association between p38 and FOXN3 was also strengthened in cells overexpressing p27 (Figure 4G), inducing p38‐mediated phosphorylation of FOXN3 residues S83 and S85, followed by FOXN3 dissociation from hnRNPU and subsequent degradation (Figure 4H,I), consequently leading to the increased degradation of IκBα (Figure 4I). Consistent with this finding, depletion of p27 impeded p38 binding to FOXN3, resulting in the inhibition of FOXN3 release from hnRNPU (Figure S4B). To investigate the regulatory role of the SKP2–p27 axis in modulating FOXN3 stability, we silenced p27 in SKP2‐KO MEFs and conducted quantitative PCR analysis. Our findings revealed that the depletion of p27 inhibited the increased phosphorylation of FOXN3 at S83 and S85 induced by SKP2 KO (Figure S4C). This inhibition ultimately resulted in a significant elevation of FOXN3 protein levels (Figure S4C) and subsequent transcriptional inactivation of the noncanonical NF‐κB signaling pathway (Figure 3H,I). Additionally, we identified the region within p27 required for its association with p38 or hnRNPU; our deletion‐mapping assay revealed that the C‐terminal region of p27 (aa 96–198) is primarily responsible for its association with the p38/hnRNPU complex (Figure S4D). Based on these findings, we concluded that p27 facilitates the recruitment of p38 to hnRNPU, thereby promoting FOXN3 phosphorylation. This phosphorylation event triggers the subsequent degradation of FOXN3, which in turn activates noncanonical NF‐κB signaling.

### 2.4 p27 Is Indispensable for SKP2‐Mediated Suppression of Pulmonary Inflammatory Injury

**FIGURE 5:**
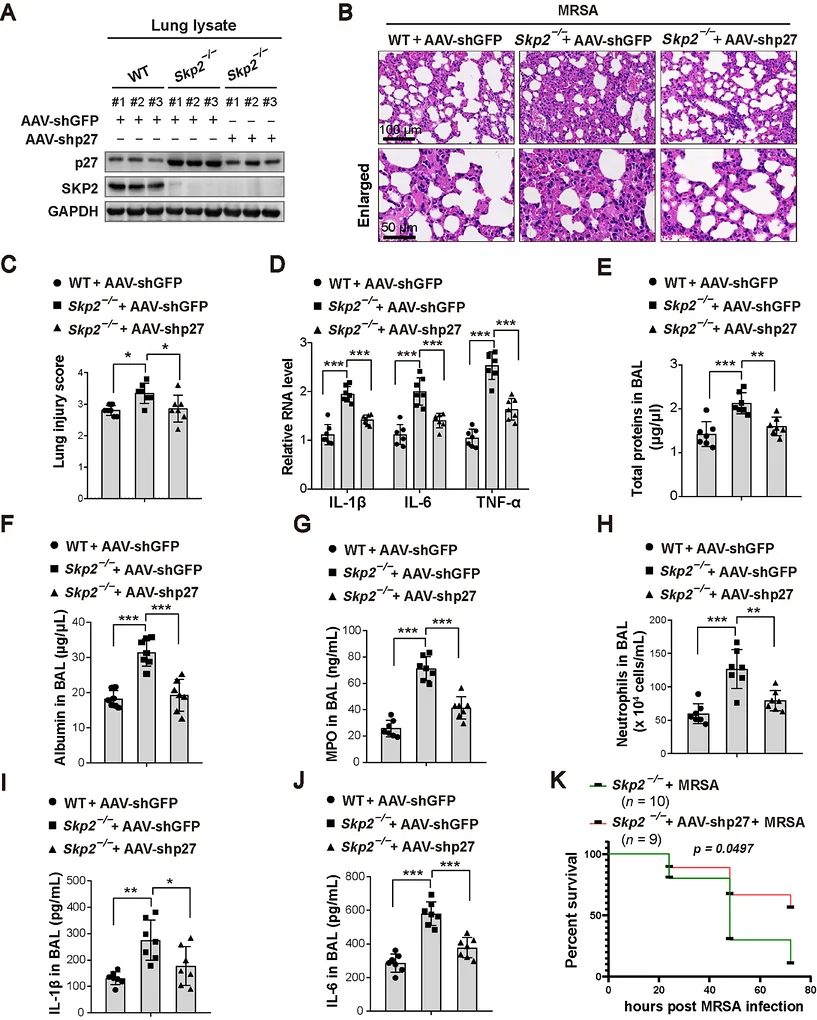
p27 depletion restrains SKP2 KO‐induced pulmonary inflammatory injury. (A) Immunoblot analysis was performed to detect the protein levels of p27 in the lung lysates of WT and SKP2 KO mice with or without AAV‐mediated p27 depletion in the presence of MRSA (*n* = 3). **(B)** H&E histological staining of lung sections from WT or SKP2 KO mice with or without AAV‐mediated p27 depletion in the presence of MRSA. **(C)** Scoring of pulmonary inflammatory injury shown in Figure 5B (*n* = 7). (D) Quantitative PCR analysis was performed to measure the levels of proinflammatory factors in the lungs of WT or SKP2 KO mice with or without AAV‐mediated p27 depletion in the presence of MRSA (*n* = 7). (E) Bicinchoninic acid (BCA) analysis of total protein concentrations in BAL fluid from WT or SKP2 KO mice with or without AAV‐mediated p27 depletion in the presence of MRSA (*n* = 7). (F) ELISA was performed to measure the levels of albumin in BAL fluid from WT or SKP2 KO mice with or without AAV‐mediated p27 depletion in the presence of MRSA (*n* = 7). (G) Neutrophil enzyme MPO analysis was performed to detect MPO activity in BAL fluid from WT or SKP2 KO mice with or without AAV‐mediated p27 depletion in the presence of MRSA (*n* = 7). (H) Neutrophil counts in BAL fluid from WT or SKP2 KO mice, with or without AAV‐mediated p27 depletion, were analyzed via Giemsa staining in the presence of MRSA (*n* = 7). (I and J) ELISA was performed to measure the level of IL‐1β (I) or IL‐6 (J) in BAL fluid from WT or SKP2 KO mice with or without AAV‐mediated p27 depletion in the presence of MRSA (*n* = 7). (K) Survival rates of SKP2‐KO mice, both with and without p27 depletion, were monitored for 72 h following MRSA infection. The data in C–J were assessed via one‐way ANOVA. All values are shown as the means ± SDs. ns, not significant; *p < 0.05, **p < 0.01, and ***p < 0.001 for comparisons between the indicated groups.

As p27 is essential for SKP2‐mediated suppression of the noncanonical NF‐κB cascade, we speculated that SKP2 might also potentially require p27 to inhibit lung inflammation and injury. To experimentally test this hypothesis, we generated an adeno‐associated virus (AAV) vector expressing a shRNA targeting p27 and delivered it, or a GFP shRNA control, via intratracheal instillation to stably silence p27 in the pulmonary tissue of SKP2‐KO mice (Figure 5A). Histological analysis indicated that depletion of p27 could limit SKP2‐KO‐induced neutrophil influx and alveolar wall thickening (Figure 5B,C). Furthermore, p27 knockdown significantly diminished the MRSA‐triggered stimulation of proinflammatory factor expression in SKP2‐KO mice (Figure 5D). We also extracted BAL fluid from MRSA‐infected SKP2‐KO mice expressing p27 shRNA or GFP shRNA control virus and found that both total alveolar protein and total albumin permeability were markedly lower than those in MRSA‐infected SKP2‐KO mice expressing AAV‐shGFP control (Figure 5E,F). In line with these results, MPO activity, neutrophil count, and the secretion of proinflammatory factors in BAL fluid were lower in p27‐depleted SKP2‐KO mice than in GFP shRNA‐treated SKP2‐KO control mice infected with MRSA (Figure 5G–J). We subsequently assessed the survival rates of SKP2‐KO mice, both with and without p27 depletion, following MRSA infection. Notably, our results demonstrated that p27 knockdown improved the survival of SKP2‐KO mice challenged with MRSA (Figure 5K). These collective findings strongly support the conclusion that p27 serves as the downstream effector protein in the SKP2‐mediated suppression of pulmonary inflammatory injury.

### 2.5 The p27–FOXN3 Axis Is Significantly Correlated With the Pathological Process of Lung Injury Triggered by MRSA Infection

Given the critical role of p27 in regulating pulmonary inflammation by inducing phosphorylation‐dependent degradation of FOXN3 at S83 and S85 during MRSA infection, we conducted further analysis to assess the correlation between p27 levels and the phosphorylation status of FOXN3 at S83 and S85 with the pathological processes of MRSA infection‐induced pulmonary inflammatory injury in vivo. Our findings revealed increased p27 levels and enhanced phosphorylation of FOXN3 at Ser83 and Ser85, accompanied by decreased levels of total FOXN3, in mice subjected to MRSA challenge (Figure S5), suggesting a potential link between the p27–FOXN3 axis and MRSA‐induced pulmonary inflammatory injury.

## 3 Discussion

SKP2 is functionally involved in several physiological and pathological processes through its E3 ligase activity, which mediates the degradation of downstream effectors such as p27, FOXO1, and PDCD4 [27, 36, 37]. The findings of this study show that SKP2 plays a vital role as a suppressor of pulmonary inflammation during MRSA infection by reducing the stability of its direct target p27, thereby activating noncanonical NF‐κB signaling in an hnRNPU‐dependent manner. Our investigations clearly demonstrate that the KO of SKP2 exacerbates the pathological process of MRSA infection‐induced pulmonary inflammatory injury. However, the inflammatory lesions resulting from SKP2 disruption were improved upon depletion of the downstream effector protein, p27. p27 is widely recognized for its ability to inhibit CDKs by reducing their levels, thereby impeding DNA replication and cell cycle progression [22, 38]. Although several studies have linked p27 with NF‐κB signaling [33, 34, 39], very little mechanistic evidence has emerged to clarify their relationship. The results of this study illustrate the crucial role of the SKP2–p27 axis in the coordinated regulation of a noncanonical NF‐κB signaling cascade during the pulmonary inflammatory response to MRSA infection. However, as an E3 ubiquitin ligase, SKP2 targets numerous substrates beyond p27, including FOXO1 and PDCD4 [27, 37, 40], both of which have been previously reported to play critical roles in pulmonary inflammatory injury [41, 42, 43]. Thus, its effects in inflammatory models cannot be fully explained by p27 degradation alone. Moreover, recent work has implicated SKP2 in lung injury via ubiquitination of the NLRP3 inflammasome [45]. Accordingly, focusing exclusively on the p27–FOXN3 axis may overlook the broader regulatory circuitry of SKP2 in vivo. Future investigations should more comprehensively delineate the downstream effectors of SKP2 in pulmonary inflammatory injury.

**FIGURE 6:**
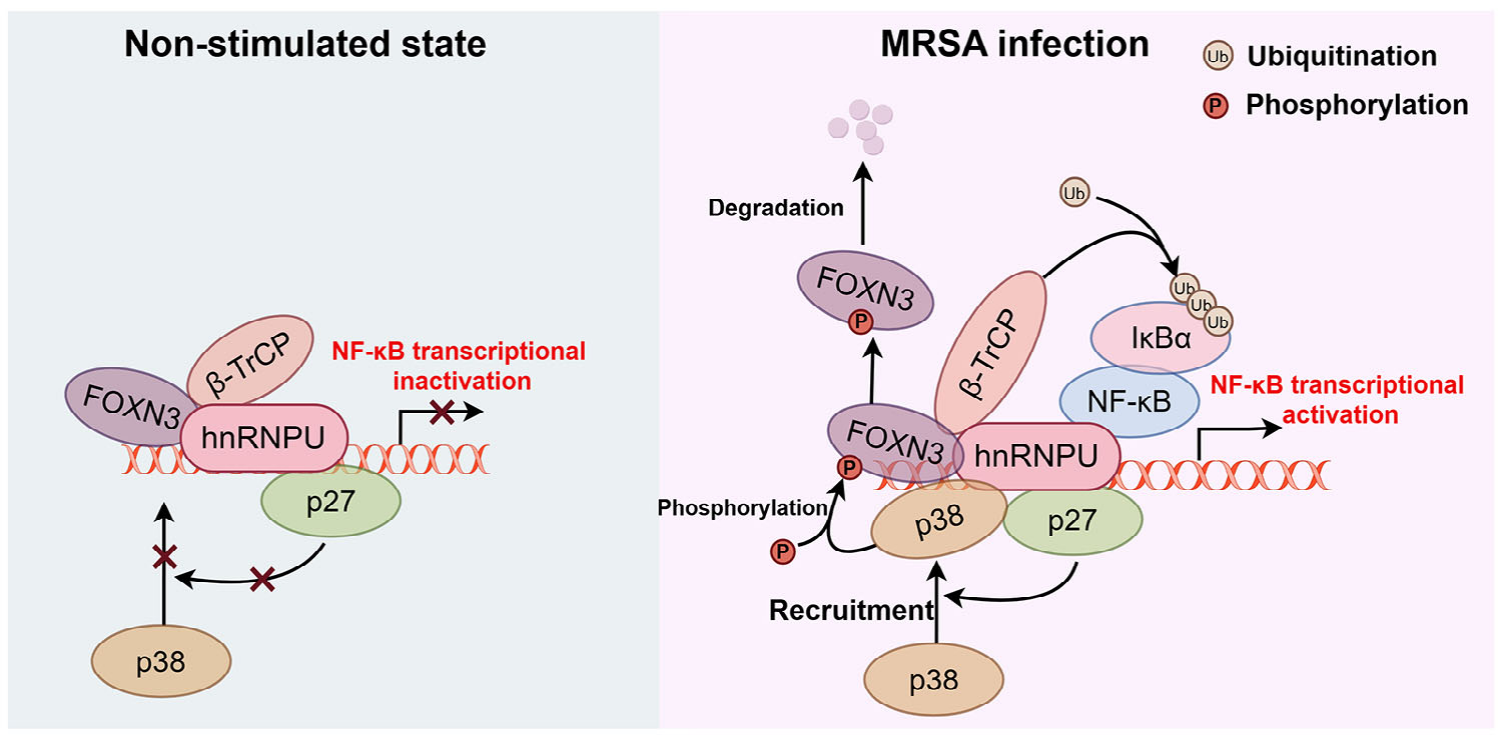
A proposed working model for the activation of NF‐κB signaling in response to MRSA via the p27‐FOXN3 axis.

Increasing evidence indicates that p27 not only serves as a G1/S gatekeeper through CDK inhibition but also participates in a wide variety of other cellular and pathological processes [24, 45]. Functionally, p27 serves as an anchor or scaffold in the assembly of regulatory complexes, especially in signal transduction [46]. In our study, we discovered that p27 facilitates the recruitment of p38 to hnRNPU through a currently unknown mechanism. In this process, p27 serves as a docking site for p38, enabling its interaction with hnRNPU. This interaction facilitates p38‐mediated phosphorylation of FOXN3 at the S83 and S85 sites, resulting in FOXN3 degradation and thereby promoting NF‐κB signaling activation (Figure 6). These combined discoveries underscore the multifaceted roles of p27 in the assembly of different regulatory modules and cellular structures.

Our previous studies revealed that hnRNPU is an essential scaffold protein in p38‐mediated FOXN3 phosphorylation, which in turn triggers phosphorylation‐dependent degradation of FOXN3 [20]. The results of the deletion mapping experiments in this study clearly indicate that p27 specifically binds to the middle region of hnRNPU and that ablation of this region blocks SKP2‐mediated p27 degradation, demonstrating that hnRNPU is indispensable for SKP2‐mediated p27 stability. On the basis of these observations, hnRNPU may exert regulatory functions in other biological processes controlled by the SKP2–p27 axis, possibly including processes such as tumorigenesis, metabolism, or cell cycle control. Further studies are needed to investigate the potential involvement of hnRNPU in these processes and understand the molecular mechanisms by which it operates within the SKP2–p27 axis. These findings open new avenues for research into the broader implications of this axis in various cellular processes and diseases.

Importantly, our in vivo studies revealed that disruption of p27 improved the decreased survival rate of these mice following SKP2 KO. Therefore, targeting the p27–FOXN3 axis presents a promising therapeutic strategy for acute pulmonary inflammatory disorders caused by bacterial infections, such as acute respiratory distress syndrome and sepsis, particularly in cases involving drug‐resistant MRSA.

## 4 Materials and Methods

### 4.1 Cell Culture

Human BEAS‐2B and HEK293T cells, purchased from the American Type Culture Collection (ATCC), were cultured in DMEM, and A549 cells were cultured in F‐12K medium. *Skp2^−/−^* MEFs, isolated from E13.5 mouse embryos, were also maintained in DMEM. All media were supplemented with 10% fetal calf serum (Gibco) and 1% penicillin–streptomycin (Millipore), and cells were cultured at 37°C in a humidified 5% CO_2_ incubator.

### 4.2 Antibodies and Chemical Reagents

The following primary antibodies were used: anti‐α‐Tubulin (11224‐1‐AP, Proteintech), anti‐hnRNPU (14599‐1‐AP, Proteintech), anti‐SKP2 (15010‐1‐AP, Proteintech), anti‐IκBα (10268‐1‐AP, Proteintech), anti‐HA (51064‐2‐AP, Proteintech), anti‐Myc (16286‐1‐AP, Proteintech), anti‐p27 (25614‐1‐AP, Proteintech), anti‐Flag (20543‐1‐AP, Proteintech), anti‐β‐actin (66009‐1‐lg, Proteintech), anti‐GAPDH (2118, Cell Signaling Technology), anti‐rabbit IgG (30000‐0‐AP, Proteintech), anti‐IKKβ (8943, Cell Signaling Technology), anti‐p38 (8690, Cell Signaling Technology), anti‐β‐TrCP (4394, Cell Signaling Technology), and anti‐FOXN3 (1:500, ab129453, Abcam). Anti‐Flag magnetic beads (A36798) and anti‐hemagglutinin (HA) magnetic beads (88836) were obtained from Thermo Fisher. The chemical reagents cycloheximide (HY‐12320) and MG132 (HY‐13259) were purchased from MedChemExpress.

### 4.3 Plasmid Constructions

Flag/HA‐tagged FOXN3, SKP2, hnRNPU, and p27 were generated by cloning human complementary DNAs into the pLV‐EF1α lentiviral vector. Flag/Myc‐tagged IκBα and Myc‐β‐TrCP, along with Flag‐, HA/Myc‐tagged p38 and IKKβ, were constructed using the pCMV6 vector. Truncation mutants of hnRNPU (1–238, 238–550, and 550–818) were generated by inserting the corresponding PCR‐amplified fragments into the pLV‐EF1α backbone. Additionally, Flag‐tagged hnRNPU deletion constructs (Δ1–238, Δ238–550, and Δ550–818) were generated using the COP‐QuickChange (COP‐QC) as described previously [47]. All plasmid constructs were confirmed by Sanger sequencing.

### 4.4 Lentiviral and AAV‐Mediated Overexpression or RNA Knockdown

To generate lentiviral particles for infection, HEK293T cells were cotransfected with either a pLV‐EF1α‐based core vector expressing the protein of interest or a pHBLV‐U6‐based lentiviral vector encoding the specific shRNA, along with the packaging plasmids psPAX2 and pMD2.G. The detailed procedure was carried out as previously described [47, 48], and the sequences of the shRNAs used were as follows: shSKP2, 5’‐GATAGTGTCATGCTAAAGAAT‐3’; shp27, 5’‐GCGCAAGTGGAATTTCGATTT‐3’. For in vivo knockdown of p27 in mouse lungs, an AAV serotype 6 system was used. Two combined shRNAs targeting p27 were cloned into the pHBAAVU6 AAV core vector, with the following target sequences: 5’‐CCCGGTCAATCATGAAGAACT‐3’ and 5’‐CGCAAGTGGAATTTCGACTTT‐3’. For virus production, HEK293A cells were cotransfected with this AAVbased core vector along with the pAAVRC6 and pHelper packaging plasmids. AAV particles were then harvested and administered via intratracheal instillation, following established protocols [20]. For siRNAmediated knockdown, cells were transfected with specific siRNAs using RNAFit transfection reagent (HBRF1000; HANBIO, Shanghai, China). The siRNA targeting sequences were as follows: sihnRNPU, 5’‐AGATCATGGCCGTGGATATTT‐3’; siSKP2, 5’‐GATAGTGTCATGCTAAAGAAT‐3’; and sip27, 5’‐GCGCAAGTGGAATTTCGATTT‐3’.

### 4.5 Immunoprecipitation Coupled With Ubiquitination Analysis

Cells intended for immunoprecipitation were harvested and lysed in a buffer composed of 150 mM NaCl, 50 mM Tris‐HCl (pH 7.4), 1% Triton X‐100, and 1 mM DTT and supplemented with both protease and phosphatase inhibitor cocktails. The resulting lysates were then incubated with the specified antibodies to perform immunoprecipitation, after which the precipitates were examined via immunoblotting. Separately, for the ubiquitination assay, cells were lysed in a buffer containing Tris‐HCl (pH 7.5), 1% SDS, 0.5 mM EDTA, and 1 mM DTT. These lysates were boiled for 10 min, then diluted 10‐fold with 50 mM Tris‐HCl (pH 7.5) buffer. After clarification by centrifugation, the recovered supernatants were processed for immunoprecipitation and subsequent immunoblot assay.

### 4.6 Animal Studies

Male C57BL/6 mice (8–10 weeks old) were used throughout this study. SKP2 KO mice were generated using the CRISPR/Cas9 system as previously described [20]. Two guide RNAs—gRNA1 (CCGTAATGGCAAGGGTCTCAGGG), targeting the forward strand adjacent to exon 3, and gRNA2 (TCAACGTCTAGTGATCACTAAGG), targeting the reverse strand adjacent to exon 6—were employed to disrupt the SKP2 gene. Successful KO was confirmed by PCR‐based genotyping. All animal procedures were approved by the Institutional Review Board of Bengbu Medical University and performed in strict compliance with relevant ethical regulations for animal research.

### 4.7 Histological Assays

Lung tissues were fixed in 4% PFA, paraffin‐embedded, and cut into 4‐µm sections, which were then stained with H&E following established protocols [20, 49, 50].

### 4.8 High‐Throughput mRNA Sequencing

mRNA sequencing was performed by GENEWIZ (Suzhou, China). In brief, total RNA was extracted using TRIzol reagent, and sequencing libraries were constructed following the standard Illumina protocol. Paired‐end sequencing was carried out on the Illumina NovaSeq 6000 platform. Subsequent data analysis was performed as previously described [52]. Raw data are available in the SRA (accession: SAMN32537730).

### 4.9 Mass Spectrometry

MS analysis was carried out on a Q Exactive system (Thermo Fisher) with a Nanospray Flex source. Peptides were trapped and separated on C18 columns via a ChromXP Eksigent system, following established protocols [20]. Data processing was assisted by Oebiotech (Shanghai). Raw data are available via ProteomeXchange/PRIDE (PXD052608). For reviewer access, the data can be viewed at: https://www.ebi.ac.uk/pride/review‐dataset/ab4e5a3acd8b4828b44db1557dc4e4d6.

Project accession: PXD052608

Token: h3XKkBHWjk9L

### 4.10 Intratracheal Administration of AAV and MRSA

MRSA (ATCC 43300) was grown in Luria–Bertani (LB) medium at 37°C to mid‐log phase, pelleted, and resuspended in PBS. Mice received intratracheal instillation of ∼50 µL containing 2–5 × 10^8^ colony‐forming units (CFU) of MRSA and were euthanized 24–48 h later. Combined AAV/MRSA delivery followed published methods [20].

### 4.11 Bronchial Alveolar Lavage (BAL) Fluid Acquisition and Analysis

To assess pulmonary inflammation and injury, BAL fluid was harvested by three sequential lung washes with 0.8 mL of ice‐cold PBS. The recovered lavage samples were centrifuged at 4°C to pellet cellular debris, and the resulting supernatants were analyzed for total protein content using a BCA assay, as well as for the proinflammatory cytokines IL‐1β and IL‐6 using ELISA. All analytical procedures followed previously described methods [20].

### 4.12 Quantitative Real‐Time PCR Analysis

Total RNA was extracted with TRIzol and reverse‐transcribed (1 µg RNA) using Vazyme RT SuperMix (R323‐01). qPCR was performed with SYBR Master Mix (Q711‐02, Vazyme), and relative expression was calculated by the 2^‐ΔΔCT^ method. Primer sequences are provided in Supporting Information Tables S1 and S2.

### 4.13 Luciferase Assay

HEK293T cells were cotransfected with a Firefly luciferase reporter plasmid driven by the ICAM1 or CCL2 promoter, together with a Renilla luciferase reporter plasmid for normalization, as previously described [53]. NF‐κB transcriptional activity was assessed by measuring luminescence.

### 4.14 ChIP Assay

Cells (1 × 10^6^) were crosslinked with 1% formaldehyde (10 min, room temperature), quenched with 125 mM glycine, and lysed in ChIP lysis buffer. Genomic DNA was sonicated to 100–500 bp fragments. Cleared lysates were immunoprecipitated with indicated antibodies, and enriched DNA was analyzed by quantitative PCR assay. All procedures were performed as previously described [54]. Primer sequences are provided in Supporting Information Table S3.

### 4.15 MRSA In Vitro Infection Experiment

Approximately 80% of confluent cells were exchanged with fresh invasion medium before MRSA infection. The MRSA bacteria, which were cultured overnight, were collected by centrifugation at 4°C for 10 min at 5000 × g, followed by two washes prior to incubation with the MRSA strain. The cells were then infected at an MOI (multiplicity of infection) of 20:1. After 3 h of infection, the infected cell cultures were washed three times and treated with gentamicin to eliminate any remaining extracellular bacteria. The infected cells were washed again and subsequently lysed for further analysis.

## Statistical Analysis

All data were validated by three independent replicates. Statistical analysis was performed with GraphPad Prism. Two‐group comparisons used two‐tailed Student's *t*‐tests, and multi‐group comparisons (≥ 3 groups) used one‐way ANOVA with Tukey's post hoc test. Significance was set at *p* < 0.05.

## Author Contributions

W.L., Y.Z., C.Z., and E.J.M. designed and conceived the project. Y.L. contributed to the methodology. Jinjin Yu, X.H., H.W., and Jingyu Yu performed most of the experiments. L.J., J.Z., and X.H. performed the data analysis. X.Z. and E.J.M. wrote the manuscript. All the authors commented on the manuscript.

## Funding

This study was funded by the National Natural Science Foundation of China (82402634 to J.Y.), the Natural Science Foundation of Anhui Province (2508085Y055 to X.Z.), the Scientific Research Project of Higher Education Institutions in Anhui Province (2024AH051236 to J.Z.), Excellent Research and Innovation Team of the Anhui Provincial Department of Education (2025AHGXZK10019 to W.L.), and the Translational Project in Clinical Medical Research in Anhui Province (202304295107020074 to Y.Z.; 202304295107020081 to W.L.). The funders had no involvement in study design, data analysis, manuscript preparation, or publication decisions.

## Ethics Statement

All experimental protocols involving animals, as well as in vitro MRSA infection procedures, received formal approval from the Institutional Review Board of Bengbu Medical University (Approval No. 2023‐679) and were conducted under biosafety level 2 conditions. The study complied with all relevant ethical regulations governing animal research and in vitro MRSA infection experiments.

## Conflicts of Interest

The authors declare no conflicts of interest.

## Supporting information




**Supporting File**: mco270963‐sup‐0001‐SuppMat.docx.

## Data Availability

All data are presented in the manuscript, figures, and supplemental files. RNA‐Seq data are available in the SRA (SAMN32537730), and mass spectrometry proteomics data are accessible via ProteomeXchange/PRIDE (PXD052608).
