# Supplementary material for: SKP2 Regulates Pulmonary Inflammatory Injury Triggered by Bacterial Infection Through p27‐Mediated Degradation of FOXN3
Source: MedComm (2020). 2026 Sep 11;7(9):e70963. doi: 10.1002/mco2.70963 (PMC13570262; doi:10.1002/mco2.70963)
Supplement: Supplementary file 1 [file MCO2-7-e70963-s001.docx]

**Supplementary information**

**SKP2 Regulates Pulmonary Inflammatory Injury Triggered by Bacterial Infection through p27-Mediated Degradation of FOXN3**

**This file includes:**

Supplementary Figures S1-S5.

Supplementary Table S1-S3.

Figure S1. p27 positively regulates NF-κB signaling activation by controlling IκBα stability.

Figure S2. p27 is a downstream regulator of the SKP2-mediated NF-κB signaling cascade.

Figure S3. HnRNPU is required for SKP2-mediated p27 degradation and NF-κB activation.

Figure S4. Domain Mapping of the p27/p38/hnRNPU Complex Interaction.

Figure S5. The levels of FOXN3 phosphorylation at S83 and S85, as well as p27, were elevated following MRSA infection.

Table S1. The primer sequences for quantitative PCR analysis (for humans).

Table S2. The primer sequences for quantitative PCR analysis (for mice).

Table S3. The primer sequences for ChIP-quantitative PCR analysis.

Figure S1

**Figure S1. p27 positively regulates NF-κB signaling activation by controlling IκBα stability. Related to Figure 3**

**(A)** A ubiquitination assay was conducted to assess the impact of p27 on Flag-tagged IκBα ubiquitination in HEK293T cells, both with and without p27 knockdown, in the presence or absence of MRSA infection (MOI, 20). Prior to collection, the cells were treated with MG-132 (20 μM, 4 h).

**(B)** Immunoblot analysis was performed to examine the effect of lentivirus-mediated p27 depletion on IκBα protein levels in BEAS-2B cells with or without MRSA treatment (MOI, 20).

**(C)** A chomatin immunoprecipitation assay was performed to examine the effect of p27 on p65 binding affinity with its target gene promoters in BEAS-2B cells with or without lentivirus-mediated p27 knockdown in the presence of MRSA infection (MOI, 20).

**(D)** Quantitative PCR analysis was performed to detect the effect of p27 on NF-κB transcriptional activation in BEAS-2B cells with or without lentivirus-mediated p27 knockdown in the presence or absence of MRSA infection (MOI, 20).

**(E)** Immunoblot analysis was performed to detect the effect of gradually increasing p27 protein levels on Myc-tagged IκBα expression in HEK293T cells.

**(F)** Immunoblot analysis was performed to examine the effect of lentivirus-mediated p27 overexpression on IκBα protein levels in BEAS-2B cells in the presence or absence of MRSA infection (MOI, 20).

**(G)** A ubiquitination assay was conducted to investigate the impact of p27 overexpression on Flag-tagged IκBα ubiquitination in HEK293T cells. The cells were transfected with the specified combinations and subjected to MRSA infection (MOI, 20) or left uninfected. Prior to collection, the cells were treated with MG-132 (20 μM, 4 h).

Data C and D were assessed via one-way ANOVA and are presented as the means ± SDs. ***P* < 0.01, and ****P* < 0.001 for comparisons between the indicated groups.

Figure S2

**Figure S2. p27 is a downstream regulator of the SKP2-mediated NF-κB signaling cascade. Related to Figure 3**

**(A)** Immunoblot analysis was performed on siSKP2 or sip27-mediated knockdown ATII cells subjected to MRSA infection (MOI, 20) for 24 hours.

**(B)** Quantitative PCR analysis showing the RNA expression levels of proinflammatory factors in ATII cells treated with siRNA against SKP2 or against both SKP2 and p27, following MRSA infection (MOI, 20, 24 hrs).

**(C)** A chromatin immunoprecipitation assay was conducted to assess the binding affinity of p65 to its target gene promoters in ATII cells treated with siRNA against SKP2 or against both SKP2 and p27, following MRSA infection (MOI, 20) for 24 hours.

The B and C data were assessed by one-way ANOVA and are shown as the means ± SDs. ***P* < 0.01 and ****P* < 0.001 for comparisons between the indicated groups.

Figure S3

**Figure S3. HnRNPU is required for SKP2-mediated p27 degradation and NF-κB activation. Related to Figure 4**

**(A)** Anti-Flag IP was performed to define the region within hnRNPU responsible for its interaction with p27 in HEK293T cells. The indicated truncation peptides of hnRNPU were cotransfected with p27 into HEK293T cells for IP.

**(B)** Immunoblot analyses were performed on both BEAS-2B and ATII cells with or without hnRNPU knockdown, following MRSA infection (MOI, 20) prior to collection.

**(C)** Quantative PCR analyses were performed on both BEAS-2B and ATII cells with or without hnRNPU knockdown, following MRSA infection (MOI, 20) prior to collection.

**(D)** Immunoblot analysis was performed to examine the effect of hnRNPU overexpression on p27 protein levels in HEK293T cells transfected with the indicated combinations. Prior to collection, the cells were treated with MRSA infection (MOI, 20).

**(E)** A ubiquitination assay was performed to examine the effect of hnRNPU overexpression on SKP2-mediated p27 ubiquitination in HEK293T cells. The cells were treated with MG-132 (20 μM, 4 h) before collection.

**(F)** Anti-Flag IP was performed to detect the effect of p27 on the interaction between IKKβ and hnRNPU in HEK293T cells transfected with the indicated combinations.

**(G)** Anti-Myc IP was performed to detect the effect of p27 on the interaction between β-TrCP and hnRNPU in HEK293T cells transfected with the indicated combinations.

The C data was assessed by a two-tailed Student's t-test and is shown as the means ± SDs. ***P* < 0.01 and ****P* < 0.001 for comparisons between the indicated groups.

Figure S4

**Figure S4. Domain Mapping of the p27/p38/hnRNPU Complex Interaction. Related to Figure 4**

**(A)** Anti-Flag immunoprecipitation (IP) in HEK293T cells was performed to assess the effect of deleting the middle region (amino acids 238–550) of hnRNPU on its interaction with p27 and p38. WT or the Δ238–550 truncated mutant of hnRNPU was co-transfected with HA-tagged p27 and p38 into HEK293T cells for IP analysis.

**(B)** Anti-Flag IP was performed to detect the effect of p27 depletion on the interaction between FOXN3 and p38 or hnRNPU in HEK293T cells transfected with the indicated combinations of HA-tagged p38, Flag-tagged FOXN3, and shp27. Prior to collection, the cells were treated with MG-132 (20 μM, 2 hr).

**(C)** Immunoblot analysis was conducted in WT and SKP2-KO MEFs, with or without p27 knockdown, to assess the impact of p27 depletion on the indicated proteins.

**(D)** Anti-Flag IP in HEK293T cells was performed to identify the region of p27 responsible for its interaction with hnRNPU and p38. WT p27, or its N-terminal (amino acids 1–95) or C-terminal domain (amino acids 96–198), was co-transfected with HA-tagged hnRNPU or p38 into HEK293T cells for IP analysis. The red asterisk indicates the target band.

Figure S5

**Figure S5. The levels of FOXN3 phosphorylation at S83 and S85, as well as p27, were elevated following MRSA infection.**

Immunoblot analysis was performed to evaluate the levels of FOXN3 phosphorylation at S83 and S85, as well as the total p27 protein in lung lysates obtained from mice, both with and without MRSA administration.

**Table S1.** The primer sequences for quantitative PCR analysis (for humans).

| IL-1β-F | TGCCCGTCTTCCTGGGAGGG |
| --- | --- |
| IL-1β-R | GGCTGGGGATTGGCCCTGAA |
| IL-6-F | CCTCTTCAGAACGAATTGAC |
| IL-6-R | GTTCAGGTTGTTTTCTGCCAG |
| TNF-α-F | GAGGCCAAGCCCTGGTATG |
| TNF-α-R | CGGGCCGATTGATCTCAGC |
| CCL2-F | CCAGCAGCAAGTGTCCCAAAG |
| CCL2-R | TGCTTGTCCAGGTGGTCCATG |
| GAPDH-F | CATGAGAAGTATGACAACAGCCT |
| GAPDH-R | AGTCCTTCCACGATACCAAAGT |

**Table S2.** The primer sequences for quantitative PCR analysis (for mice).

| IL-1β-F | TGCCACCTTTTGACAGTGATG |
| --- | --- |
| IL-1β-R | TGATGTGCTGCTGCGAGATT |
| IL-6-F | CCACTTCACAAGTCGGAGGCTTA |
| IL-6-R | CCAGTTTGGTAGCATCCATCATTTC |
| CCL2-F | CAGCACCAGCACCAGCCAAC |
| CCL2-R | TCTGGCTGAGCCAACACGTG |
| TNF-α-F | TATGGCCCAGACCCTCACA |
| TNF-α-R | GGAGTAGACAAGGTACAACCCATC |
| GAPDH-F | AGGTCGGTGTGAACGGATTTG |
| GAPDH-R | TGTAGACCATGTAGTTGAGGTCA |

**Table S3.** The primer sequences for ChIP-quantitative PCR analysis.

| CCL2-F | AGTTGCCTGATCTATAACATG |
| --- | --- |
| CCL2-R | GCTCATTCAAATGCAGAATAG |
| IL-6-F | TTGCCATGCTAAAGGACGTC |
| IL-6-R | GACTCATGGGAAAATCCCAC |
| TNF-α-F | ACTGAAACCAGCATTATGAG |
| TNF-α-R | TGGGTGGGGGTAGCAGGGAC |
